# Development of a portable and cost-effective femtosecond fibre laser synchronizable with synchrotron X-ray pulses

**DOI:** 10.1107/S1600577524003667

**Published:** 2024-06-20

**Authors:** Keisuke Kaneshima, Takumi Kyoda, Shuta Sugeta, Yoshihito Tanaka

**Affiliations:** a Graduate School of Science University of Hyogo 3-2-1 Koto Kamigori, Ako Hyogo678-1297 Japan; RIKEN SPring-8 Center, Japan

**Keywords:** femtosecond fibre lasers, pulse synchronization, time-resolved spectroscopy, portable laser system

## Abstract

This study introduces a compact, portable femtosecond fibre laser system designed for synchronization with SPring-8 synchrotron X-ray pulses in a uniform filling mode. Unlike traditional titanium–sapphire mode-locked lasers, which are fixed installations, our system utilizes fibre laser technology to provide a practical alternative for time-resolved spectroscopy, striking a balance between usability, portability and cost-efficiency. Comprehensive evaluations, including pulse characterization, timing jitter and frequency stability tests revealed a centre wavelength of 1600 nm, a pulse energy of 4.5 nJ, a pulse duration of 35 fs with a timing jitter of less than 9 ps, confirming the suitability of the system for time-resolved spectroscopic studies. This development enhances the feasibility of experiments that combine synchrotron X-rays and laser pulses, offering significant scientific contributions by enabling more flexible and diverse research applications.

## Abstract 2

This study introduces a compact, portable femtosecond fibre laser system optimized for synchronization with SPring-8 synchrotron X-ray pulses in a uniform filling mode, offering a practical and cost-effective alternative to traditionally, fixed, installed laser systems for time-resolved spectroscopy combining synchrotron X-ray pulses and laser pulses.

## 1. Introduction

The synchronization of synchrotron X-ray pulses with laser pulses is crucial for advancing experimental science, especially in time-resolved spectroscopy (Mizutani *et al.*, 1997 ▸; Tanaka *et al.*, 2000 ▸). While advancements in high-order harmonic generation techniques using high-intensity lasers have made it possible to generate soft X-ray pulses beyond the water window (Spielmann *et al.*, 1997 ▸; Popmintchev *et al.*, 2012 ▸; Ishii *et al.*, 2014 ▸; Fu *et al.*, 2020 ▸), accessing photon energies exceeding 1 keV remains challenging with laser based methods. Synchronization of synchrotron X-rays with lasers enables experiments that are difficult to carry out with lasers alone, such as X-ray pump or probe time-resolved spectroscopy (Pfeifer *et al.*, 2006 ▸; Yamamoto & Matsuda, 2013 ▸; Milne *et al.*, 2014 ▸), and the development of innovative light sources through the interaction between accelerated electrons and laser electric fields (Albert *et al.*, 2010 ▸, Jochmann *et al.*, 2013 ▸; Tanaka *et al.*, 2023 ▸). With the advent of X-ray free-electron lasers (XFELs) (Emma *et al.*, 2010 ▸; Ishikawa *et al.*, 2012 ▸; Ackermann *et al.*, 2007 ▸; Allaria *et al.*, 2012 ▸), the importance of synchronization techniques has further increased. Techniques such as balanced optical cross correlators (Schibli *et al.*, 2003 ▸; Kim *et al.*, 2008 ▸; Song *et al.*, 2011 ▸) are being developed for synchronization with femtosecond-order XFEL pulses (Schulz *et al.*, 2015 ▸). Although combining XFELs and lasers enables ultrafast time-resolved studies, the role of synchrotron and laser combinations remains significant. This is because synchrotron radiation offers a broad spectrum and high flux, high repetition rates, a wide wavelength selection and easier access to beam time, facilitating a wide range of experiments that are complementary to those possible with XFELs (Brynes, 2021 ▸). Consequently, the development of time-resolved measurement systems utilizing synchrotron X-rays is continuously advancing (Fukaya *et al.*, 2022 ▸).

To conduct time-resolved experiments, somewhat more complex procedures are required than for experiments using only X-rays. Traditional laser systems, exemplified by titanium–sapphire mode-locked lasers, are typically complex and costly, often requiring extensive infrastructure and lacking portability. They are usually installed at specific beamlines and local alignment can be a time-consuming task, consuming a significant portion of the limited beam time available.

Fibre lasers offer an effective solution to these challenges. They are compact, lightweight and economical to construct. Their design facilitates easy resonator length adjustments through simple processes like fusion splicing to extend or cutting to shorten the fibre, which allows for synchronization with varying repetition frequencies of X-ray pulses at different synchrotron facilities and operating modes. However, fibre lasers have limitations, such as being more susceptible to temperature fluctuations affecting the repetition frequency, particularly when the resonator comprises long fibre lengths. They also have lower energy per pulse, making them less suitable as pump sources. Despite these drawbacks, a femtosecond fibre laser capable of synchronizing with synchrotron X-ray pulses is quite useful for applications in synchrotron X-ray pump and laser-probe experiments.

We report the successful development of a compact, portable femtosecond fibre laser system, specifically designed for synchronization with SPring-8 A-mode operation, with a repetition frequency of 42.38 MHz (http://www.spring8.or.jp/en/users/operation_status/schedule/several_bunch_mode). Through evaluation, including measurements of timing jitter and assessments of long-term stability of the repetition frequency and the output power, we confirm that our laser system achieves synchronization with synchrotron radiation at the desired accuracy. Fibre lasers are already utilized as clocks in synchrotron facilities (Kim & Song, 2016 ▸), but this study develops a fibre laser with pulse energies of several nanojoules, directly usable for optical experiments. This development enhances time-resolved studies with synchrotron light sources, offering improved access to advanced spectroscopy and better integration with various experimental setups.

## 2. Design and characterization of the femtosecond fibre laser

**Figure 1:**
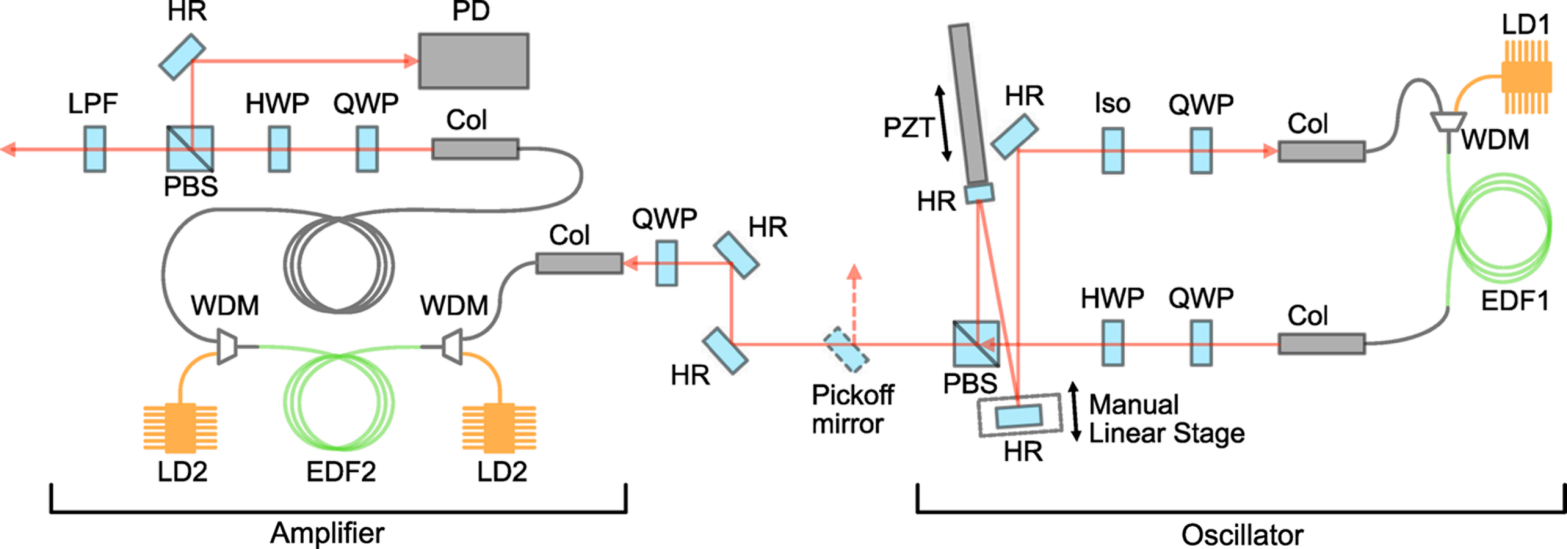
Schematic of the developed fibre laser system. LD1: laser diode (3S photonics, 1999CHP), WDM: wavelength division multiplexer, EDF1: erbium-doped fibre (Coractive, EDF-L1500), Col: collimator, QWP: quarter wave plate, HWP: half wave plate, Iso: Faraday isolator (Thorlabs, IO-4–1550-VLP), HR: dielectric mirror, PZT: piezo electric transducer (Thorlabs, PK4FXP2), PBS: polarization beam splitter, LD2: laser diode (3S photonics, 1999CVB), EDF2: erbium-doped fibre (Liekki, Er80-8/125), PD: photodetector (Thorlabs, DET08C), LPF: low-pass filter (Thorlabs, FEL1500).

Fig. 1 ▸ is a schematic of the developed fibre laser system. The system is primarily divided into the oscillator and amplifier sections. All the optical components, along with the LD drivers, are mounted on a 400 mm × 600 mm breadboard, facilitating easy portability of the entire setup.

**Figure 2:**
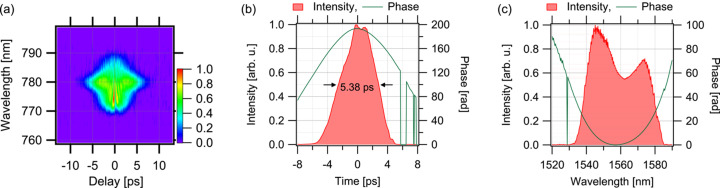
Results of the SHG-FROG measurement of the oscillator output pulses. (*a*) Experimentally obtained FROG trace. (*b*) Retrieved temporal profiles (intensity and phase). (*c*) Retrieved spectral profiles (intensity and phase).

The oscillator features passive mode-locking enabled by nonlinear polarization rotation (Matsas *et al.*, 1992 ▸; Tamura *et al.*, 1992 ▸) with a configuration that allows for resonator length adjustment via a manual linear stage and a piezo-electric transducer (PZT). The method for timing synchronization using the PZT is discussed in the following section. The oscillator has a ring cavity, and, considering synchronization with X-ray pulses during SPring-8 A-mode operation, its repetition frequency is set at 42.38 MHz. The gain medium for the laser is an erbium-doped fibre (EDF). Although Yb-doped fibres offer superior energy efficiency, Er-doped fibres have advantages in terms of cost, short-pulse operation and broadband capabilities (Brida *et al.*, 2014 ▸). Furthermore, Er-doped fibre lasers enable probing in longer wavelength regions that are difficult to access with Ti:sapphire lasers. Additionally, generating the second harmonic of the Er-doped fibre laser output yields wavelengths similar to those of Ti:sapphire lasers, allowing for the reuse of optical systems designed for Ti:sapphire applications. For the oscillator, an EDF (Coractive, EDF-L1500) with a length of 3.03 m is used as the gain medium. The EDF is forwardly pumped using a laser diode (LD) (3S Photonics, 1999CHP). The output wavelength of the LD is 980 nm and operated at a current of 700 mA, producing an approximate output power of 400 mW. The length of the other silica single-mode fibre constituting the oscillator is approximately 1.54 m, and the free-space section is 0.35 m long. The dispersion of EDF-L1500 is approximately −25 ps nm^−1^ km^−1^ at a wavelength of 1550 nm. Considering Corning’s SMF-28 as the single-mode fibre, its dispersion is around 18 ps nm^−1^ km^−1^ at a wavelength near 1550 nm (https://www.corning.com/opticalfiber). The overall cavity exhibits positive dispersion and is operated in the dissipative soliton mode-locking regime (Chong *et al.*, 2006 ▸; Grelu & Akhmediev, 2012 ▸). The average output power of the oscillator was 60 mW, which was achieved after using a low-pass filter to remove the pump laser diode components. The output pulses from the oscillator were characterized by second-harmonic generation frequency-resolved optical gating (SHG-FROG) (Trebino, 2000 ▸) using a nonlinear crystal of BiB_3_O_6_ with a thickness of 0.5 mm (Newlight Photonics, BiTC7050), and the results are presented in Fig. 2 ▸. Fig. 2 ▸(*a*) shows the raw experimental data of the FROG trace, and Figs. 2 ▸(*b*) and 2 ▸(*c*) display the retrieved intensity and phase information in the temporal and spectral domains, respectively. The quadratic phase dependence in both domains indicates the presence of a linear chirp in the pulse. Though SHG FROG cannot determine the sign of the chirp, the cavity dispersion suggests that it is a positive chirp. Additionally, the shape of the spectral intensity is characteristic of the dissipative soliton mode-locking regime. The full width at half-maximum (FWHM) of the pulse duration was 5.38 ps [Fig. 2 ▸(*b*)]. This temporally stretched pulses are spectrally broadened and temporally compressed in the amplifier section.

**Figure 3:**
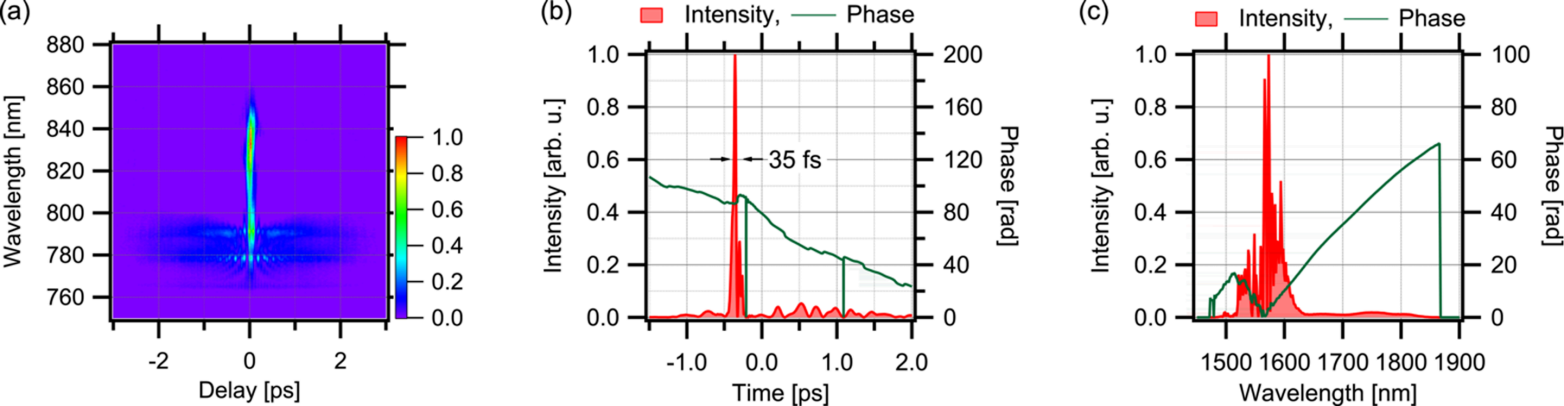
Results of the SHG-FROG measurement of the amplifier output pulses. (*a*) Experimentally obtained FROG trace. (*b*) Retrieved temporal profiles (intensity and phase). (*c*) Retrieved spectral profiles (intensity and phase).

A quarter-wave plate placed just before the amplifier input collimator serves to control the occurrence of nonlinear optical effects through the dispersion of power into different polarization modes. In the amplifier, a 0.53 m length of erbium-doped fibre (Liekki, Er80-8/125) is used, preceded by 2.51 m of silica single-mode fibre and followed by 7.57 m of silica single-mode fibre (Corning, SMF-28). The EDF is bidirectionally pumped by two LDs (3S Photonics, 1999CVB). The output wavelength of the LDs is 980 nm. Each LD operates at a current of 1200 mA, producing an approximate output power of 700 mW. With the EDF dispersion being 19.6 ps nm^−1^ km^−1^ (Sun *et al.*, 2016 ▸), the amplifier as a whole exhibits negative dispersion. This, combined with the nonlinear optical effects in the fibre, serves to cancel the positive dispersion of the output light from the oscillator and achieves pulse compression. The length of the fibre following the EDF was adjusted while monitoring the degree of pulse compression. Within the amplifier, nonlinear optical effects cause polarization rotation, resulting in components that cannot be fully converted back to linear polarization. At the amplifier’s output, a polarization beam splitter separates such components, which are then used as a signal to synchronize the repetition frequency. The average output power was approximately 190 mW, corresponding to a pulse energy of 4.5 nJ, which was achieved after using a low-pass filter to remove the pump laser diode components. Fig. 3 ▸(*a*) presents the raw experimental data of the FROG trace for the amplifier output pulses. Figs. 3 ▸(*b*) and 3 ▸(*c*) illustrate the retrieved intensity and phase information in the temporal and spectral domains, respectively. As can be seen from the FROG trace, the shorter wavelength components below 1600 nm are not fully compressed. The pulse duration, measured at the intensity FWHM, is 35 fs, but the aforementioned uncompressed components form a pedestal. For applications where such a pedestal is a concern, using a low-pass filter to cut the shorter wavelength components may yield cleaner pulses with a smaller pedestal. However, in synchronization experiments with synchrotron radiation, the pedestal is likely to be a non-issue since the temporal duration of the synchrotron radiation X-ray pulses is several tens of picoseconds (Khan, 2016 ▸), which is relatively long. Nevertheless, reducing the pulse duration to 35 fs is advantageous for applications in nonlinear optical measurements, such as surface second harmonic generation (SHG) probing, where a higher peak intensity is beneficial. Given its intended use as a probe light, we focused on optimizing pulse duration and spectral width. When pulse energy is crucial, generally, larger pulse energies can be attained by extending the pulse duration.

## 3. Synchronization with synchrotron X-ray pulses

Repetition frequency monitoring by the photodetector (PD) and resonator length feedback control via a PZT (Thorlabs, PK4FXP2, displacement 57.5 µm) allows for synchronization with an external reference signal (Hall *et al.*, 2001 ▸). The displacement range of the PZT is an important design parameter, as it determines the magnitude of the compensable deviation in repetition frequency of the oscillator. The repetition frequency of the developed oscillator is matched to SPring-8 A-mode operation frequency and is set at 42.38 MHz, which is relatively low for a mode-locked fibre laser (implying a longer resonator length). Considering that the resonator length is *l* and its change induced by the PZT is ±δ*l*, the corresponding change in the repetition frequency of the resonator can be approximated as 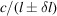 ≃ 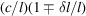. From this relationship, it follows that a lower repetition frequency requires a larger PZT displacement. In our case, the resonator comprises 4.5 m of fibre. Given the thermal coefficient of delay for the optical fibre at 35.7 ps km^−1^ K^−1^ (Hartog *et al.*, 1979 ▸), a 1 K change in ambient temperature would result in a frequency shift of approximately 290 Hz for a fibre oscillator operating near 42.38 MHz. Therefore, as a guideline, to compensate for the frequency variation due to a ±1 K change in ambient temperature, a PZT displacement of 50 µm is required, which corresponds to a total resonator length change of 100 µm (accounting for the round trip). Hence, the developed system incorporates a PZT with a displacement of 57.5 µm to adequately compensate for temperature variations of approximately ±1 K.

**Figure 4:**
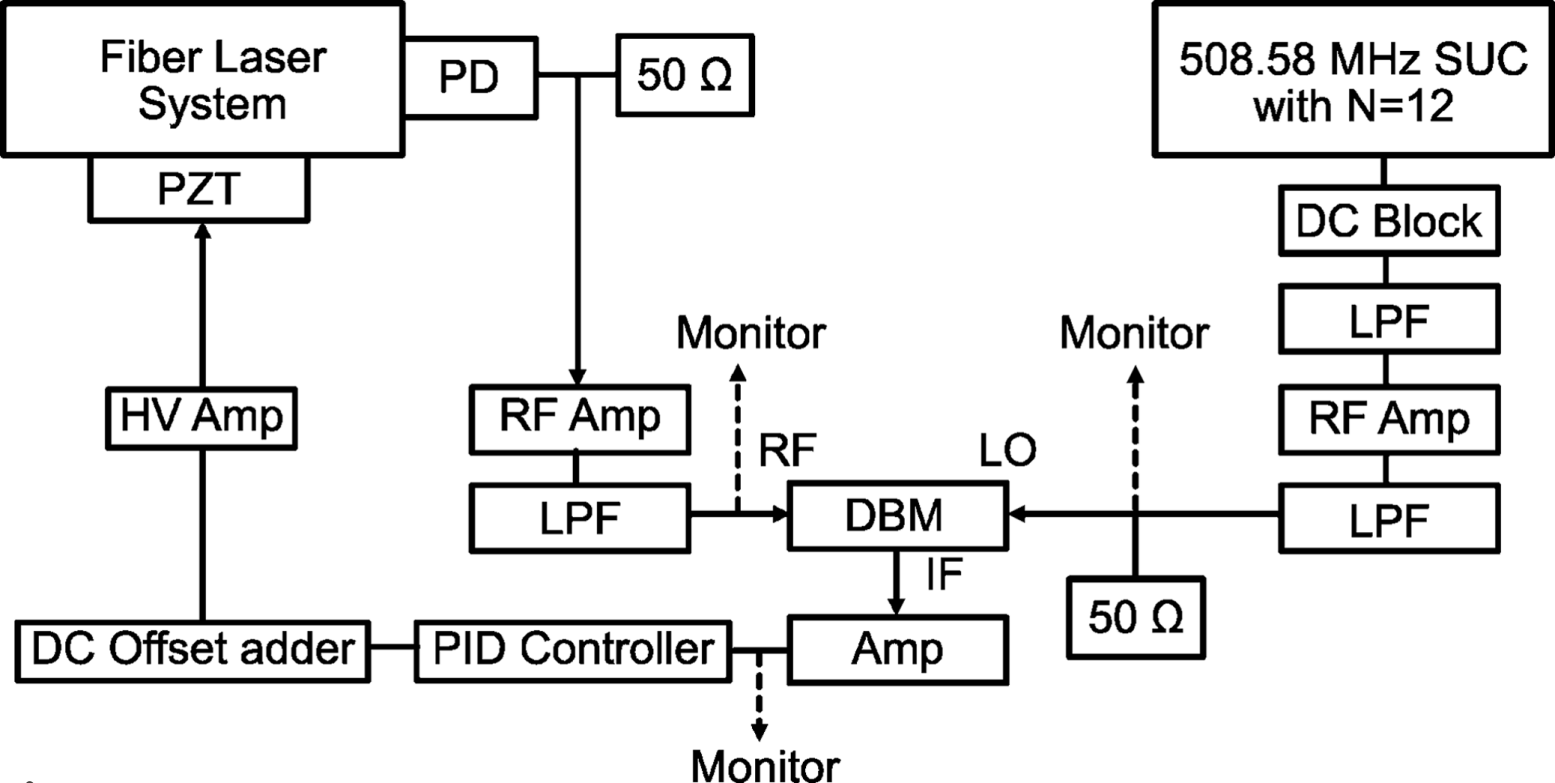
Schematic of the signal processing chain for timing synchronization. PD: photodetector (Thorlabs, DET08C), 50 Ω: 50 Ohm terminator, RF Amp: radio-frequency power amplifier (Mini-Circuits, ZFL-1000LN+), LPF: low-pass filter (Crystek, CLPFL-0050), DBM: double balanced mixer (Mini-Circuits, ZX05-1-S+), DC Block: DC block filter (Thorlabs, EF500), HV amp: high-voltage amplifier (Thorlabs, MDT694B), PZT: piezoelectric transducer (Thorlabs, PK4FXP2).

Fig. 4 ▸ depicts the signal processing chain for timing synchronization in the developed system. While state-of-the-art techniques using a balanced optical cross correlator have been reported to enable synchronization on the order of femtoseconds or less (Schibli *et al.*, 2003 ▸; Kim *et al.*, 2008 ▸; Schulz *et al.*, 2015 ▸; Togashi *et al.*, 2020 ▸), the pulse duration of synchrotron X-ray pulses is relatively long, *e.g.* several tens of picoseconds (Khan, 2016 ▸). Therefore, in this study, we opted to use an electrical signal-based phase look loop only for synchronization. The PD signal is filtered to the fundamental frequency by a low-pass filter (LPF) and then input into the RF input of a double balanced mixer (DBM). For the reference signal, SPring-8 utilizes a 508.58 MHz synchronous universal counter (SUC) at each beamline, capable of generating a reference signal synchronized with the X-ray pulses (Suzuki *et al.*, 1999 ▸). In our experiment, a signal with a division ratio of 12 was generated, corresponding to 42.38 MHz, to synchronize with the X-ray pulse timing during SPring-8 A-mode operation. The SUC signal, a logic signal varying between 0 V and −1 V, is cleansed of DC offset and further monochromated by an LPF before being fed into the LO input of the DBM. The difference frequency between the RF and LO signals, emerging from the IF output of the DBM, is used as an error signal for feedback control. For feedback, we use a PID controller mode in a field-programmable gate array (FPGA)-based multi-measurement device (Liquid Instruments, Moku:Go). The output from this PID controller, ranging from −5 V to +5 V, is adjusted to a 0 V to 10 V range using a custom DC offset adder, then amplified by a high-voltage (HV) amplifier with a gain of 15 to produce a voltage output ranging from 0 V to 150 V for PZT control.

**Figure 5:**
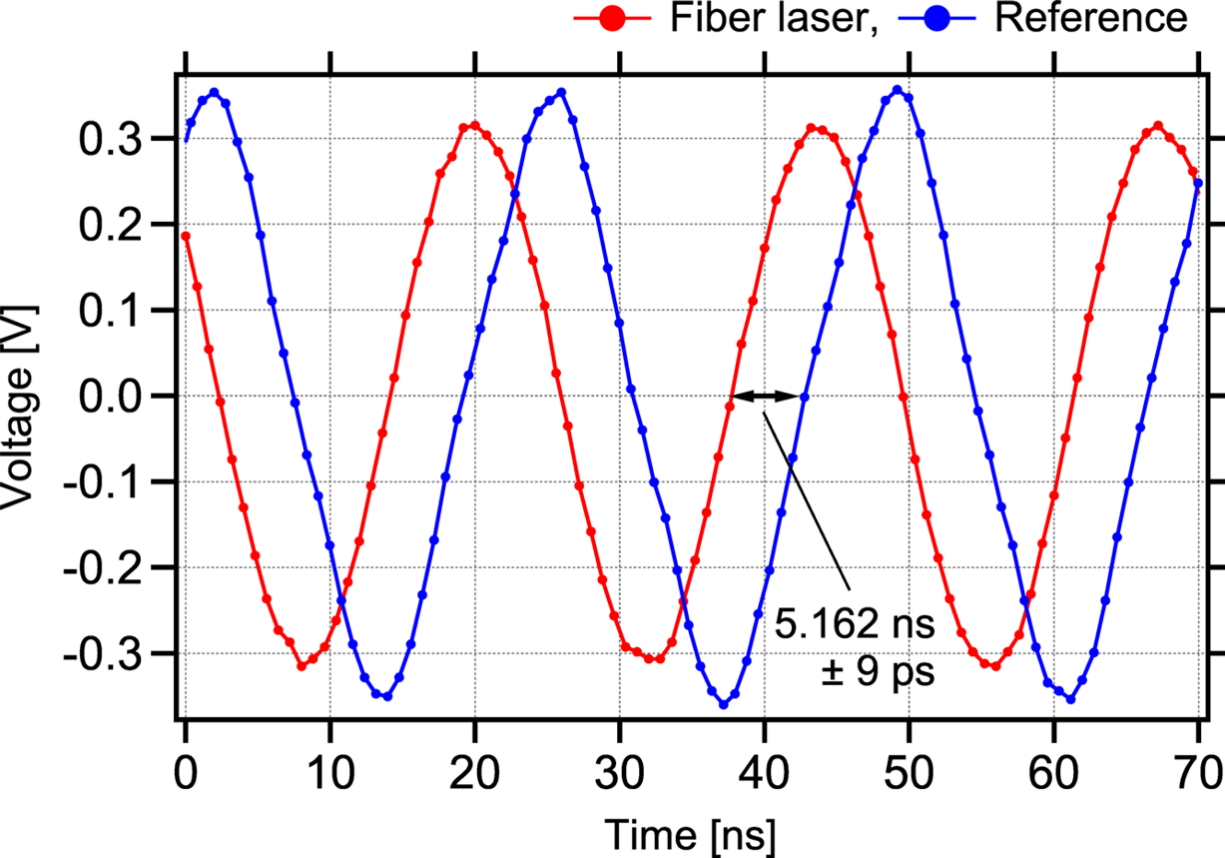
Timing jitter measurement between the fibre laser and the reference signals using an oscilloscope (Tektronix, DPO71604).

To verify the capability of timing synchronization in practice, we brought our fibre laser to SPring-8 and synchronized it with the reference signal supplied by SPring-8’s SUC, conducting measurements of timing jitter. During this experiment, the fibre laser was placed on a standard workbench, not specifically designed for optical applications, outside the experimental hutch at SPring-8. We used the automatic measurement function of a 16 GHz-bandwidth oscilloscope (Tektronix, DPO71604) to figure out the timing jitter, measuring the delay between the mid amplitude points of the fibre laser and the reference signals (Fig. 5 ▸). To enhance the accuracy of the measurements, we tolerated waveform clipping and magnified the vertical (voltage) axis on the oscilloscope. As a result, the timing jitter between the signals was determined to be approximately 9 ps in standard deviation. Given that the time resolution of the oscilloscope is 20 ps (50 GS s^−1^), this 9 ps measurement represents the maximum possible jitter. Thus, the true jitter is likely to be less. In the case of SPring-8, the temporal duration of X-ray pulses is 33 ps (FWHM) (Yabashi *et al.*, 2002 ▸). Given that the measured jitter is smaller than the X-ray pulse duration, this level of precision is sufficient for time-resolved spectroscopy applications combining synchrotron X-rays and laser pulses.

**Figure 6:**
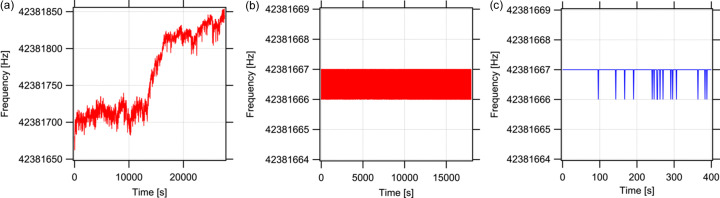
Results of the repetition frequency measurement using a frequency counter with a time constant of 1 s. Repetition frequency of the fibre laser (*a*) without synchronization and (*b*) with synchronization. (*c*) Reference signal of the synchronization (output of the 508.58 MHz SUC with a frequency division factor of 12).

To evaluate long-term stability, we used a frequency counter (AKIZUKI DENSHI TSUSHO, AE-FCOUNT3) equipped with a 12.8 MHz ±1 p.p.m. oscillator (Mercury, VM39S5G). This experiment was also conducted in a similar setting to the timing jitter measurement, with the fibre laser placed on a workbench outside the experimental hutch at SPring-8. Fig. 6 ▸(*a*) shows the temporal variation of the laser repetition frequency without synchronization, exhibiting drift that is likely due to changes in ambient temperature. Fig. 6 ▸(*b*) shows the frequency when synchronized to the reference, with measured repetition frequencies only alternating between 42381666 Hz and 42381667 Hz. Fig. 6 ▸(*c*) reveals that the frequency measurement results for the reference source also fluctuate solely between these two values, demonstrating that the fibre laser signal is indeed synchronized with the reference. The observed frequency fluctuation is likely attributable to the accuracy limit of the frequency counter. The synchronization is maintained over 5 h, and the synchronization remained uninterrupted until it was deliberately disengaged.

## 4. Power stability and beam quality

**Figure 7:**
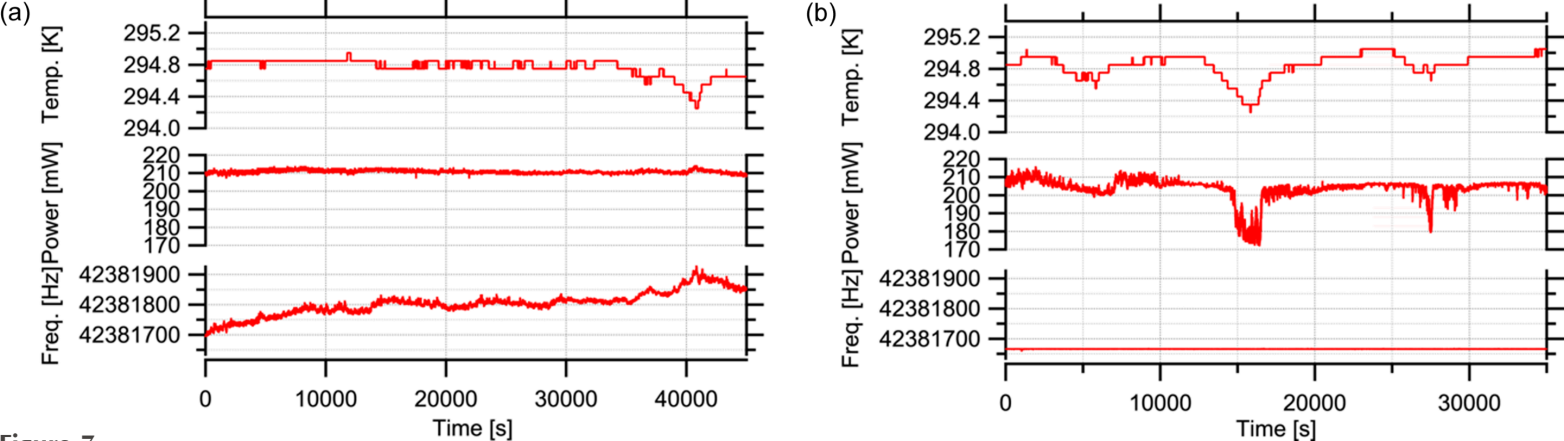
Results of simultaneous measurements of the temperature near the fibre system, the laser output power and the laser repetition frequency in a laboratory environment: (*a*) without synchronization and (*b*) with synchronization.

We conducted additional laboratory experiments to check the long-term output power stability. Fig. 7 ▸ presents the results where the temperature near the fibre system as well as the output power and repetition frequency of the laser were simultaneously measured. These tests evaluated the system stability with the phase-locked loop (PLL) feedback control both activated and deactivated under varying ambient temperature conditions. The data indicate that, despite fluctuations in ambient temperature, the fibre laser maintained frequency synchronization. However, an observation was made that, while the output energy generally remains stable, it can fluctuate depending on temperature. This fluctuation is attributed to the system being composed of standard (non-polarization maintaining) fibre – important from a cost perspective – and the output being linearly polarized. Changes in temperature can affect the polarization state of the optical pulses, leading to variations in output energy. For applications sensitive to pulse energy, it is advisable to monitor output power and calibrate experimental data accordingly.

**Figure 8:**
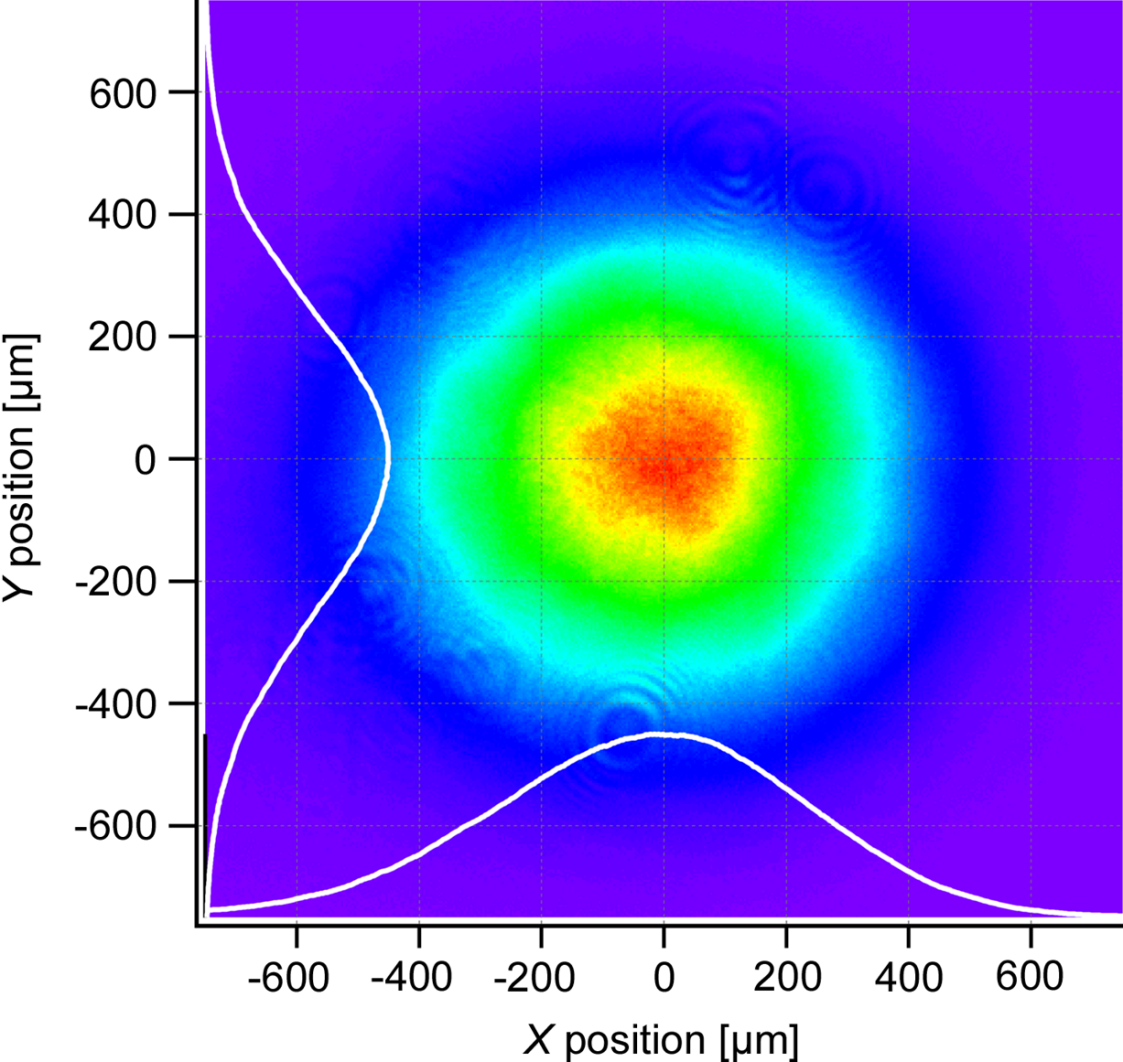
Beam profile of the second harmonic of the fibre laser output beam.

For the spatial profile of the beam, we utilized an Si CMOS camera (Thorlabs, CS165MU) for measurement. Due to the limited sensitivity of standard Si CMOS cameras at 1.6 µm, we employed second-harmonic generation to convert the wavelength to 800 nm. This conversion was achieved using a 0.5 mm-thick BiB_3_O_6_ crystal, enabling the beam profile measurement with the Si CMOS camera. Fig. 8 ▸ displays the results, confirming a high-quality Gaussian beam profile, attributable to the mode filtering property inherent to single-mode fibres. Furthermore, our compact system offers an advantage over fixed lasers at beamlines, which necessitate transporting the beam to the application site. Such transport can degrade the beam profile through diffraction, self-focusing and other effects. By positioning our system directly at the point of use, we ensure a pristine beam, beneficial for applications that demand precise beam focusing and high spatial resolution.

## 5. Summary

In summary, we have successfully developed a compact and portable femtosecond fibre laser system tailored for synchronization with the 42.38 MHz repetition frequency of SPring-8 A-mode operation, featuring an FWHM pulse duration of 35 fs. This system also demonstrates cost-effective synchronization solutions by achieving a timing jitter with a measured upper limit of 9 ps using a single piezoelectric transducer (PZT) and custom-made electronics, which is well below the duration of synchrotron X-ray pulses, making it highly suitable for the vast majority of time-resolved experiments utilizing synchrotron radiation. Our comprehensive evaluation, including timing jitter measurements, long-term frequency stability, output power stability and the spatial properties of the beam, confirms the system’s utility in conjunction with synchrotron X-ray pulses. The high peak power of the system, a result of its short pulse duration, enables its use for probing nonlinear optical processes, such as surface second-harmonic generation. The exceptional spatial beam quality of the system also contributes to achieving high peak intensity and is advantageous for applications requiring precise beam focusing and high spatial resolution. Additionally, its broad spectral range in the near-infrared region opens up applications such as observing photo-induced phase transition processes in materials like VO_2_, where probing at 1600 nm reveals changes more readily than at 800 nm (Currie *et al.*, 2017 ▸). The second overtones of C—H stretching, O—H stretching and N—H stretching vibrations within this NIR range offer opportunities for observing changes in various organic molecules following X-ray excitation. Furthermore, light around 1.6 µm meets the phase-matching conditions for efficient terahertz generation using organic nonlinear optical crystals like DAST (4-*N*,*N*-di­methyl­amino-4′-*N*′-methyl-stilbazolium tosyl­ate) (Jazbinsek *et al.*, 2019 ▸). Utilizing this property allows for the development of a portable terahertz wave source synchronized with synchrotron radiation.

Overall, this system marks an advancement for the scientific community, providing a cost-effective solution that enhances accessibility, thereby enabling more efficient, detailed and flexible time-resolved spectroscopic experiments requiring synchronization between synchrotron X-ray and laser pulses. This approach broadens the range of research groups able to engage in advanced time-resolved studies, breaking down barriers to entry and fostering wider participation in cutting-edge scientific investigations.
